# Comprehensive Analysis of the Value of SMYD Family Members in the Prognosis and Immune Infiltration of Malignant Digestive System Tumors

**DOI:** 10.3389/fgene.2021.699910

**Published:** 2021-07-16

**Authors:** Donghui Liu, Xuyao Wang, Enhong Shi, Liru Wang, Minghao Nie, Long Li, Qingxin Jiang, Pengyu Kong, Shuai Shi, Chao Wang, Sen Yan, Zhihui Qin, Shuang Zhao

**Affiliations:** ^1^Department of Oncology, Heilongjiang Provincial Hospital, Harbin, China; ^2^Department of Pharmacy, Harbin Second Hospital, Harbin, China; ^3^Department of Pathology, Heilongjiang Provincial Hospital, Harbin, China; ^4^Department of General Surgery, First Affiliated Hospital of Harbin Medical University, Harbin, China; ^5^Department of General Surgery, Harbin 242 Hospital of AVIC, Harbin, China; ^6^Department of Orthopedics, Second Affiliated Hospital of Harbin Medical University, Harbin, China; ^7^Department of Cardiology, The Fourth Affiliated Hospital of Harbin Medical University, Harbin, China; ^8^Department of Cardiology, Second Affiliated Hospital of Harbin Medical University, Harbin, China; ^9^Department of Cardiology, First Affiliated Hospital of Harbin Medical University, Harbin, China; ^10^Department of Obstetrics and Gynecology, The Fourth Affiliated Hospital of Harbin Medical University, Harbin, China

**Keywords:** SMYD, malignant tumors, prognosis, immune infiltration, clinical stage, TP53, gene mutation

## Abstract

**Background:**

The SET and MYND domain-containing (SMYD) gene family comprises a set of genes encoding lysine methyltransferases. This study aimed to clarify the relationship between the expression levels of SMYD family members and the prognosis and immune infiltration of malignant tumors of the digestive system.

**Methods:**

The Oncomine, Ualcan, Kaplan–Meier Plotter, cBioPortal, Metascape, and TIMER databases and tools were used to analyze the correlation of SMYD family mRNA expression, clinical stage, TP53 mutation status, prognostic value, gene mutation, and immune infiltration in patients with esophageal carcinoma (ESCA), liver hepatocellular carcinoma (LIHC), and stomach adenocarcinoma (STAD).

**Results:**

In ESCA, the mRNA expression of SMYD2/3/4/5 was significantly correlated with the incidence rate, that of SMYD2/3 with the clinical stage, that of SMYD2/3/4/5 with TP53 mutation status, that of SMYD2/4/5 with overall survival (OS), and that of SMYD1/2/3/4 with relapse-free survival (RFS). In LIHC, the mRNA expression of SMYD1/2/3/4/5 was significantly correlated with the incidence rate, that of SMYD2/4/5 with the clinical stage, that of SMYD3/5 with TP53 mutation status, that of SMYD2/3/4/5 with OS, and that of SMYD3/5 with RFS. In STAD, the mRNA expression of SMYD2/3/4/5 was significantly correlated with the incidence rate, that of SMYD1/4 with the clinical stage, that of SMYD1/2/3/5 with TP53 mutation status, that of SMYD1/3/4 with OS, and that of SMYD1/3 with RFS. Furthermore, the function of SMYD family mutation-related genes in ESCA, LIHC, and STAD patients was mainly related to pathways, such as mitochondrial gene expression, mitochondrial matrix, and mitochondrial translation. The expression of SMYD family genes was significantly correlated with the infiltration of six immune cell types and eight types of immune check sites.

**Conclusion:**

SMYD family genes are differentially expressed and frequently mutated in malignant tumors of the digestive system (ESCA, LIHC, and gastric cancer). They are potential markers for prognostic prediction and have important significance in immunity and targeted therapy.

## Introduction

Esophageal carcinoma (ESCA), liver hepatocellular carcinoma (LIHC) and gastric cancer (GC) are the main malignant tumors of the digestive system, accounting, respectively, for 5.3, 8.2, and 8.2% of cancer-related deaths worldwide (Bray et al., 2018). Therefore, their prevention and treatment should attract substantial attention. Although the development of endoscopy, imaging, and other technologies has greatly increased the detection rate of high-risk malignant digestive system tumors (Banks et al., 2019; van der Pol et al., 2019), the mortality rate remains high, because of the lack of effective early diagnostic and prognostic markers. The SET and MYND domain-containing (SMYD) gene family comprises a set of genes encoding lysine methyltransferases. SMYD family proteins have structural similarities, consisting of six similar domains from the N-terminal to C-terminal, of which SET and MYND are among the most important. To date, five SMYD family members, SMYD1–5, playing important roles in embryonic development, skeletal, and cardiac muscle development, have been found in the human genome (Donlin et al., 2012; Nestorov et al., 2015; Fujii et al., 2016). Furthermore, recent studies have shown that SMYD family members play an important role in the occurrence and development of different tumors. For example, SMYD1 mutations have been implicated in splenic marginal zone lymphoma (SMZL) (Peveling-Oberhag et al., 2015). In colorectal cancer and LIHC, SMYD3 can activate multiple signal pathways by regulating transcription, and promote, among other malignant cell phenotypes, tumor cell proliferation, invasion, and epithelial to mesenchymal cell transformation (Hamamoto et al., 2004; Sarris et al., 2016; Chen et al., 2019). In breast cancer (BC) cells, SMYD3 can regulate the cell cycle and promote cancer cell migration by combining with the cyclin A1 (CCNA1) and myosin light chain 9 (MYL9) promoters (Luo et al., 2014; Mazur et al., 2014). SMYD4 may act as an inhibitor of certain transcription factors to regulate the expression of platelet-derived growth factor receptor A (PDGFR-A), thereby inhibiting the proliferation and survival of BC cells (Hu et al., 2009). Furthermore, previous studies have shown that some SMYD family members are differentially expressed in GC and related to prognosis. For example, SMYD2 is highly expressed in GC and related to poor prognosis. The related mechanism is involved in tumor cell proliferation, migration, and invasion (Komatsu et al., 2015). The tissue expression of SMYD3 was significantly positively correlated with the expression of transforming growth factor β1 (TGF-β1) in GC, whereas the prognosis of GC patients with high SMYD3 and TGF-β1 expression was poor (Liu H. et al., 2015).

An imbalance of immune effector cells in the tumor microenvironment contributes to malignant tumor cell immune escape. In recent years, tumor immunotherapy has received extensive attention in a variety of solid tumors and has been regarded as an important treatment method (Kirkwood et al., 2012). For example, the application of programmed death 1 (PD-1) inhibitors in ESCA, advanced liver cancer, and locally advanced or metastatic GC has achieved good curative effects (Högner and Thuss-Patience, 2021; Joshi and Badgwell, 2021; Kim et al., 2021). Related studies have shown that some SMYD family members are closely related to immune infiltration (Stender et al., 2012; Nagata et al., 2015; Xu et al., 2015), but the underlying immune mechanism in tumors remains unclear.

The occurrence and development of malignant digestive tract tumors is a complex process. In the past, some studies have reported the expression pattern of SMYD family members in some cancer patients and its correlation with prognosis. However, the entire SMYD family has not been so far systematically investigated in malignant digestive tract tumors. Therefore, we conducted a comprehensive analysis of the SMYD family based on public data reposited in various large databases to determine its role in malignant digestive system tumors.

## Materials and Methods

### ONCOMINE

Oncomine^1^ is currently the world’s largest oncogene microarray database and integrated data mining platform, which integrates RNA and DNA-seq data from gene expression omnibus (GEO), The Cancer Genome Atlas (TCGA), and published literature sources. To date, ONCOMINE contains 65 gene expression datasets comprising nearly 48 million gene expression measurements form over 4700 microarray experiments. We used the cancer microarray database (without TCGA data) to analyze the mRNA expression levels of SMYD family members in ESCA, LIHC, GC, and normal esophagus, liver, and stomach tissues (Rhodes et al., 2004) and summarized the whole picture of SMYD gene family from a macro perspective. Enter SMYD1/2/3/4/5 in the “search” module in turn, and set the following thresholds: “*P*-value = 0.01,” “fold-change = 1.5,” “THRESHOLD (GENE RANK) = Top 10%,” “data type = mRNA,” output Disease Summary for SMYD family.

### UALCAN

UALCAN^2^ is a comprehensive and interactive online data analysis website based on relevant data found in TCGA database, including gene expression data of 184 ESCA, 371 LIHC, and 415 GC patients. The portal’s user-friendly features allow to perform: (1) analyze relative expression of a query gene(s) across tumor and normal samples, as well as in various tumor sub-groups based on individual cancer stages, tumor grade, race, body weight, or other clinicopathologic features, (2) estimate the effect of gene expression level and clinicopathologic features on patient survival; and (3) identify the top over-and under-expressed (up and down-regulated) genes in individual cancer types. We used the UALACAN database to evaluate the expression levels of SMYD family members in ESCA, LIHC, gastric adenocarcinoma (STAD), and normal esophagus, liver, and stomach tissues, and determine the correlation between clinical stage and TP53 status (Chandrashekar et al., 2017), and to verify the data from mRNA expression levels in Oncomine. Enter SMYD1/2/3/4/5 in the “Enter gene symbol(s)” module in turn, and then select the “expression” parts of ESCA, LIHC, and STAD, respectively, and select “Sample type,” “Individual cancer stages,” and “TP53 mutation status,” respectively in the Gene expression based on module. Considering the unequal variances, the significance of differences in the transcriptional levels was evaluated using the Student’s *t*-test, and a *P*-value of <0.05 was considered statistically significant.

### Kaplan–Meier Plotter

The Kaplan--Meier Plotter^3^ is an online database containing microarray gene expression data and survival information from public databases, such as GEO, TCGA, and the European Genome-phenome Archive (EGA), this study included 80 ESCA patients, 371 LIHC patients, and 375 GC patients, but patients miss expression values and lack complete clinical data. We divided patient samples into high- and low expression groups according to the best cut off of the expression level of SMYD family members, and used the Kaplan–Mayer Plotter to analyze the overall survival (OS) and relapse-free survival (RFS) of ESCA, LIHC, and STAD patients. Enter SMYD1/2/3/4/5 in the “Gene symbol” of the “Start KM Plotter for pan-cancer” module, select Auto select best cut off in the “Split patients by” module, select OS, RFS in the “Survival” module, and then select ESCA, LIHC, and STAD to generate survival curves, and use Kaplan–Meier method to draw survival curves. A *P*-value of <0.05 was considered statistically significant.

### cBioPortal

The cBioPortal^4^ integrates data from large-scale cancer research projects, such as TCGA and the International Cancer Genome Consortium (ICGC), whose gene data types cover somatic mutations, DNA copy number changes, mRNA and microRNA expression, DNA methylation, protein and phosphorus protein abundance, and provides visual and multidimensional cancer genomic data (Cerami et al., 2012; Gao et al., 2013). This study based on TCGA database, gene expression data of 181 ESCA, 366 LIHC, and 412 GC patients were included. We obtained the relevant module information about SMYD family gene mutations from the cBioPortal. Select ESCA, LIHC, and STAD in “Query” module, apply “TCGA, PanCancer Atlas” data set, set the parameters “mRNA Expression: mRNA expression *z*-scores relative to diploid samples (RNA Seq V2 RSEM),” “Enter a *z*-score threshold ±1.8,” “Select Patient/Case Set: Samples with mRNA data (RNA Seq V2),” enter SMYD1/2/3/4/5 in “Enter Genes” to generate a mutation frequency visualization chart, and then select the top 10 ESCA, LIHC, and STAD genes significantly related to SMYD family gene mutations in “Co-expression” module for enrichment analysis after removing duplicates.

### Metascape

Metascape^5^ is a gene list analysis tool. It integrates data from over 40 types of biological information databases for gene annotation and analysis, and provides a unique protein–protein interaction (PPI) network analysis function. We used the “Multiple Gene list” module of the Metascape tool to perform gene annotation and enrichment analyses on the genes obtained from the cBioPortal that were highly related to ESCA, LIHC, STAD, and SMYD family member mutations (Zhou et al., 2019), set the parameters “Input as species and Analysis as species: H. sapients,” select “Custom Analysis,” set the threshold in “Enrichment” module: enrichment factor “Min overlap = 3,” “*P*-value cut-off value < 0.01,” “Min enrichment >1.5” is considered statistically significant, then select Gene Ontology (GO) enriching “Biological Processes,” “Cellular Components” and “Molecular Functions” and “KEGG pathways” classification. To further capture the relationships between the terms, a subset of enriched terms were selected and rendered as a network plot, where terms with a similarity >0.3 were connected by edges. We selected the terms with the best *P*-values from each of the 20 clusters, with the constraint that there were no more than 15 terms per cluster and no more than 250 terms in total. The network was visualized using Cytoscape (Shannon et al., 2003), where each node represented an enriched term and was colored first by its cluster ID, and then by its *P*-value. For each given gene list, PPI enrichment analysis was carried out using the following databases: STRING (Szklarczyk et al., 2019), BioGrid (Oughtred et al., 2019), OmniPath (Li et al., 2017b), and InWeb_IM (Li et al., 2017b). Only physical interactions in STRING (physical score >0.132) and BioGrid were used (details). The molecular complex detection (MCODE) algorithm (Bader and Hogue, 2003) was applied to identify densely connected network components.

### TIMER

TIMER^6^ is a comprehensive resource based on the relevant data in TCGA database, including gene expression data of 184 ESCA, 371 LIHC, and 415 GC patients. Tumor progression and the efficacy of immunotherapy are strongly influenced by the composition and abundance of immune cells in the tumor microenvironment. Due to the limitations of direct measurement methods, computational algorithms are often used to infer immune cell composition from bulk tumor transcriptome profiles. TIMER2.0 provides more robust estimation of immune infiltration levels for TCGA or user-provided tumor profiles using six state-of-the-art algorithms. We mainly used modular input to evaluate the expression levels of SMYD family members in ESCA, LIHC, STAD, and evaluated six types of immune infiltrating cells and related immune check sites (Li et al., 2020, 2017a). Enter SMYD1/2/3/4/5 in the “Gene Symbol” of “Diff Exp” module to generate a block diagram of gene expression level distribution, and use Wilcoxon test to evaluate the significance of transcription level difference. A *P*-value of <0.05 is considered statistically significant. Set the parameters in the “Gene” module: “Gene Symbol: SMYD1/2/3/4/5,” “Cancer Types: ESCA, LIHC, STAD,” “Immune Infiltrates: B cells, CD8+ T cells, CD4+ T cells, macrophages, neutrophils, dendritic cells,” generate a scatter plot of immune cell infiltration correlation, which shows the purity-corrected partial Spearman’s rho value and statistical significance. A *P*-value of <0.05 is considered statistically significant. Set the parameters in the “Correlation” module: “Cancer Types enter ESCA, LIHC, STAD,” “Gene Symbols: (*Y*-axis) enter CD274, CTLA4, GZMB, HAVCR2, LAG3, PDCD1, TIGIT, TNF,” “Gene Symbols: (*X*-axis) enter SMYD1/2/3/4/5,” “Correlation Adjusted by: Tumor Purity,” generate the scatter plot of immune test site correlation, which is statistically significant using Spearman’s rho value. A *P*-value of <0.05 is considered statistically significant.

## Results

### mRNA Expression Levels of SMYD Family Members in ESCA, LIHC, GC, and Normal Esophagus, Liver, and Stomach Tissues in the Different Data Sets

**FIGURE 1:**
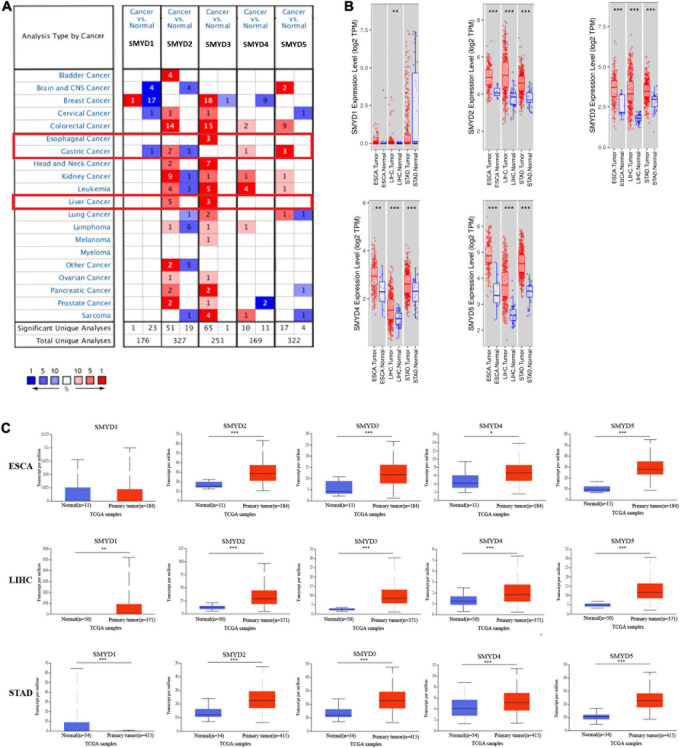
The mRNA expression levels of SMYD family members in 20 types of cancers (Oncomine). The graph shows the numbers of datasets with statistically significant mRNA high expression (red) or low expression (blue) of the target gene. The threshold was designed with following parameters: *P*-value of 0.01 and fold change of 1.5, and data type of mRNA **(A)**. The mRNA expression levels of SMYD family members in ESCA, LIHC, STAD (red), and normal tissues of esophagus, liver and stomach (blue) (Timer) (^∗^*P* < 0.05, ^∗∗^*P* < 0.01, ^∗∗∗^*P* < 0.001) **(B)**. The mRNA expression levels of SMYD family members in ESCA, LIHC, STAD (red), and normal tissues of esophagus, liver, and stomach (blue) (UALCAN) (^∗^*P* < 0.05, ^∗∗^*P* < 0.01, ^∗∗∗^*P* < 0.001) **(C)**.

We measured the mRNA expression of the five SMYD family members in 20 cancer types, and compared them with those of normal tissues using the Oncomine database (Figure 1A). According to the information from ten data sets, SMYD1 was significantly downregulated in GC patients (D’Errico et al., 2009), SMYD2 was significantly upregulated in LIHC patients (Chen et al., 2002; Wurmbach et al., 2007; Mas et al., 2009; Roessler et al., 2010), and significantly upregulated in GC patients (Chen et al., 2003; D’Errico et al., 2009; Wang et al., 2012). SMYD3 was significantly overexpressed in ESCA and LIHC patients (Wurmbach et al., 2007; Hu et al., 2010; Kim et al., 2010; Roessler et al., 2010; Su et al., 2011), whereas SMYD4 and SMYD5 were significantly overexpressed in GC patients (D’Errico et al., 2009; Wang et al., 2012; Table 1).

**TABLE 1:** Significant changes of mRNA expression levels of SMYD family members in ESCA, LIHC, STAD, and normal tissues of esophagus, liver, and stomach (Oncomine).

Types of cancer VS. normal	Fold change	*P*-value	*t*-test	Ref	PMID
**SMYD1**					
Gastric mixed adenocarcinoma	–2.320	0.003	3.705	DErrico gastric	19081245
**SMYD2**					
Hepatocellular carcinoma	2.137	1.54E-6	5.864	Roessler liver	21159642
Hepatocellular carcinoma	3.767	1.50E-5	5.409	Wurmbach liver	17393520
Hepatocellular carcinoma	1.537	1.87E-6	5.162	Mas liver	19098997
Hepatocellular carcinoma	1.791	2.68E-35	13.690	Roessler liver 2	21159642
Hepatocellular carcinoma	1.113	0.155	1.018	Chen liver	12058060
Gastric mixed adenocarcinoma	1.658	9.15E-5	4.699	Chen gastric	12925757
Gastric mixed adenocarcinoma	2.364	1.03E-4	4.868	DErrico gastric	19081245
Gastric mixed adenocarcinoma	–1.525	0.004	–2.841	Wang gastric	21132402
**SMYD3**					
Esophageal squamous cell carcinoma	3.232	3.72E-10	11.098	Hu esophagus	20955586
Esophageal squamous cell carcinoma	1.567	1.77E-11	7.451	Su esophagus 2	21385931
Barrett’s esophagus	1.553	1.53E-4	1.567	Kim esophagus	21152079
Hepatocellular carcinoma	3.580	1.50E-11	8.860	Wurmbach liver	17393520
Hepatocellular carcinoma	3.237	8.97E-56	19.479	Roessler liver 2	21159642
Hepatocellular carcinoma	2.444	2.57E-7	6.794	Roessler liver	21159642
**SMYD4**					
Gastric mixed adenocarcinoma	1.668	2.43E-4	3.872	DErrico gastric	19081245
**SMYD5**					
Gastric mixed adenocarcinoma	3.901	1.63E-9	8.553	DErrico gastric	19081245
Gastric mixed adenocarcinoma	4.106	1.08E-10	7.776	DErrico gastric	19081245
Gastric cancer	2.154	6.18E-4	3.660	Wang gastric	21132402

The mRNA expression of SMYD family members in ESCA, LIHC, STAD and normal esophagus, liver, and stomach tissues in TCGA database was analyzed using the TIMER data mining website. The expression of SMYD1 in ESCA tissues was not significantly different from that of normal tissues (*P* = 0.97E-01), but the expression of SMYD2/3/4/5 was significantly higher than that of normal tissues [SMYD2 (*P* = 2.88E-5), SMYD3 (*P* = 1.04E-4), SMYD4 (*P* = 3.40E-03), and SMYD5 (*P* = 2.75E-7)]. The expression of SMYD1/2/3/4/5 in LIHC tissues was significantly higher than that of normal tissues [SMYD1 (*P* = 8.02E-3), SMYD2 (*P* = 1.74E-18), SMYD3 (*P* = 7.59E-26), SMYD4 (*P* = 3.24E-7), and SMYD5 (*P* = 2.12E-23)], whereas SMYD1 expression in STAD tissues was not significantly different from that of normal tissues (*P* = 0.143E-01). However, SMYD2/3/4/5 expression was significantly higher than that of normal tissues [SMYD2 (*P* = 2.08E-13), SMYD3 (*P* = 5.18E-11), SMYD4 (*P* = 6.64E-04), and SMYD5 (*P* = 7.54E-21)] (Figure 1B).

The mRNA expression levels of SMYD family members in ESCA, LIHC, STAD, and normal esophagus, liver, and stomach tissues in TCGA database were analyzed using the UACLAN data mining website. SMYD1 expression in ESCA tissues was not significantly different than that of normal tissues (*P* = 1.92E-01). However, SMYD2/3/4/5 expression was significantly higher than that of normal tissues [SMYD2 (*P* < 1.39E-10), SMYD3 (*P* = 2.14E-6), SMYD4 (*P* = 2.24E-02), and SMYD5 (*P* = 3.95E-8)]. The expression of SMYD1/2/3/4/5 in LIHC tissues was significantly higher than that in normal tissues [SMYD1 (*P* < 2.82E-3), SMYD2 (*P* < 1.00E-12), SMYD3 (*P* = 1.62E-12), SMYD4 (*P* = 1.00E-12), and SMYD5 (*P* = 1.62E-12)], whereas SMYD1 expression in STAD tissues was significantly lower than that in normal tissues (*P* = 6.60E-03). Furthermore, SMYD2/3/4/5 expression was significantly higher than that in normal tissues [SMYD2 (*P* < 1.00E-12), SMYD3 (*P* = 3.00E-15), SMYD4 (*P* = 2.62E-04), and SMYD5 (*P* = 1.62E-12)] (Figure 1C). In summary, the same results were obtained using the Oncomine, Timer, and UACLAN tools.

### Correlation of the mRNA Expression Level of SMYD Family Members With the Clinical Stage and TP53 Mutation Status in Patients With ESCA, LIHC, and STAD

**FIGURE 2:**
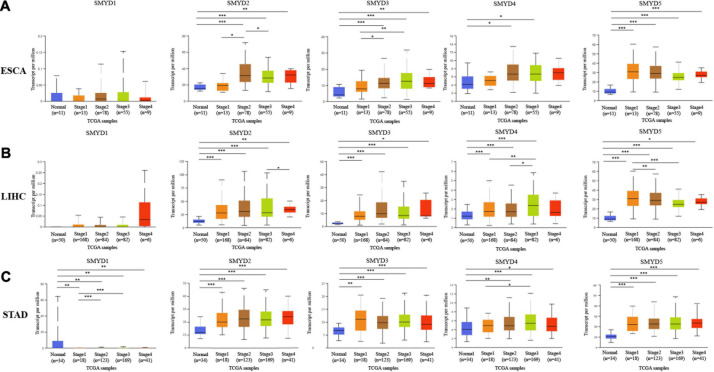
The relationship between mRNA expression levels of SMYD family members and clinical stages in ESCA, LIHC, STAD (stage-1/2/3/4 is orange, brown, green, and red, respectively), and normal tissues of esophagus, liver, and stomach (blue) (UALCAN) **(A–C)** (^∗^*P* < 0.05, ^∗∗^*P* < 0.01, ^∗∗∗^*P* < 0.001).

**FIGURE 3:**
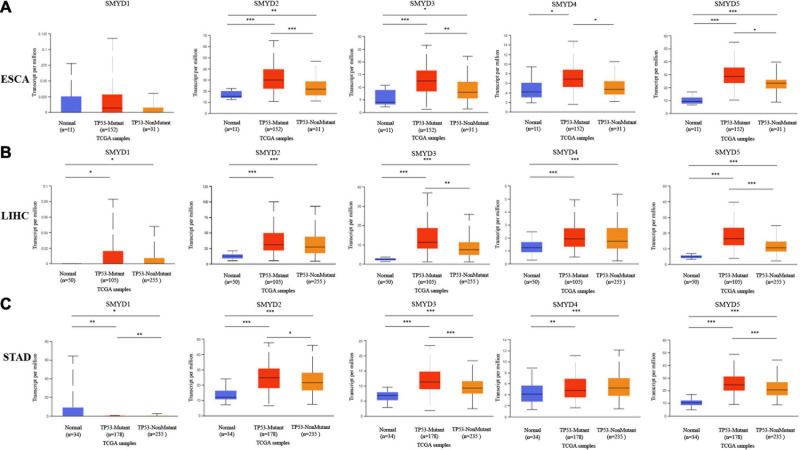
The relationship between mRNA expression levels of SMYD family members and TP53 mutation in ESCA, LIHC, STAD (mutation: red, non-mutation: orange), and normal tissues of esophagus, liver, and stomach (blue) (UALCAN) **(A–C)** (^∗^*P* < 0.05, ^∗∗^*P* < 0.01, ^∗∗∗^*P* < 0.001).

We determined the association between the mRNA expression levels of different SMYD family members and the clinical stage and TP53 mutation status of patients with ESCA, LIHC, and STAD using the UALCAN data mining website. Clinical stage correlation analysis showed significant expression differences in SMYD1 between stage-1 and stage-2, and stage-1 and stage-3 in STAD patients, in SMYD2 between stage-1 and stage-2, and stage-2 and stage-3 in ESCA patients, and between stage-3 and stage-4 in LIHC patients. Significant differences in SMYD3 expression were found between stage-1 and stage-2, and stage-1 and stage-3 in ESCA patients, and in SMYD4 between stage-1 and stage-3, stage-2 and stage-3 in LIHC patients, and between stage-1 and stage-3 in STAD patients. Significant differences in SMYD5 expression were found between stage-1 and stage-3, stage-1 and stage-3 in LIHC patients (Figures 2A–C). Notably, SMYD1 expression was significantly decreased only in the TP53 mutation group of STAD patients, whereas SMYD4 was significantly increased only in the TP53 mutation group of ESCA patients. SMYD2/3/5 expression was significantly increased in the TP53 mutation group of ESCA, LIHC, and STAD patients (Figures 3A–C), indicating that TP53 mutations may be involved in the regulation of mRNA expression of SMYD family members. Thus, in ESCA, SMYD2/3 expression was significantly related to the clinical stage, and SMYD2/3/4/5 expression to TP53 mutation status. In LIHC, SMYD2/4/5 expression was significantly related to the clinical stage, and SMYD3/5 expression was significantly related to TP53 mutation status. In STAD, SMYD1/4 expression was significantly related to the clinical stage, and SMYD1/2/3/5 to TP53 mutation status.

### The Prognostic Value of SMYD Family Members in ESCA, LIHC, and STAD Patients

**FIGURE 4:**
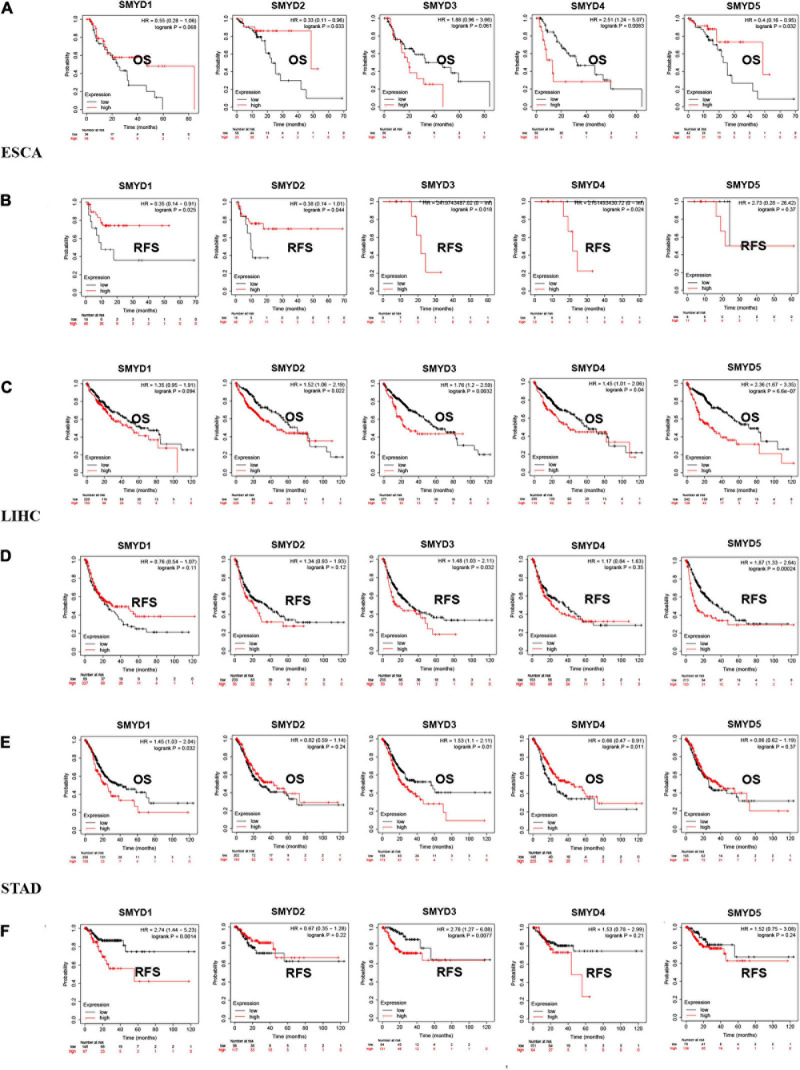
The prognostic value of mRNA expression levels of SMYD family members in ESCA, LIHC, and STAD patients (Kaplan–Meier plotter). Plot the relationship between high expression (red) and low expression (black) of target gene mRNA and OS and RFS, respectively **(A–F)** (*P* < 0.05 with statistical significance).

We analyzed the survival of different SMYD family members in ESCA, LIHC, and STAD patients using the Kaplan–Meier Plotter website and found that SMYD2/4/5 were significantly associated with OS in ESCA [SMYD2 hazard ratio (HR) 0.33 (1.11–0.96), *P* = 0.033; SMYD4 HR 2.51 (1.24–5.07), *P* = 0.0083; SMYD5 HR 0.4 (0.16–0.95), *P* = 0.032], SMYD1/2/3/4 were all significantly correlated with RFS [SMYD1 HR 0.35 (0.14–0.91), *P* = 0.025; SMYD2 HR 0.38 (0.14–1.01), *P* = 0.044; SMYD3 HR 2,419,743,487.02 (0–lnf), *P* = 0.018; SMYD4 HR 2,151,493,430.72 (0–lnf), *P* = 0.024) (Figures 4A,B). In LIHC, SMYD2/3/4/5 were significantly correlated with OS [SMYD2 HR 1.52 (1.06–2.19), *P* = 0.022; SMYD3 HR 1.76 (1.2–2.59), *P* = 0.0032; SMYD4 HR 1.45 (1.01–2.06), *P* = 0.04; SMYD5 HR 2.36 (1.67–3.35), *P* = 6.6e-07], SMYD3/5 were significantly correlated with RFS [SMYD3 HR 1.48 (1.03–2.11), *P* = 0.032; and SMYD5 HR 1.87 (1.33–2.64)], *P* = 0.00024) (Figures 4C,D). In STAD, SMYD1/3/4 were significantly correlated with OS [SMYD1 HR 1.45 (1.03–2.04), *P* = 0.032; SMYD3 HR 1.53 (1.1–2.11), *P* = 0.01; SMYD4 HR 0.66 (0.47–0.91), *P* = 0.011], SMYD1/3 were significantly correlated with RFS [SMYD1 HR 2.74 (1.44–5.23), *P* = 0.0014; and SMYD3 HR 2.78 (1.27–6.08), *P* = 0.0077] (Figures 4E,F). Overall, the SMYD gene family is closely related to ESCA, LIHC, and STAD patient prognosis. SMYD1 expression was positively correlated with ESCA prognosis and negatively correlated with STAD prognosis. SMYD2 expression was positively correlated with ESCA prognosis and negatively correlated with LIHC prognosis. SMYD3 expression was correlated with ESCA, LIHC, and STAD prognosis. SMYD4 expression was positively correlated with STAD prognosis and negatively correlated with ESCA and LIHC prognosis. Finally, SMYD5 expression was positively correlated with ESCA prognosis and negatively correlated with LIHC prognosis.

### SMYD Family Gene Mutations and Prognosis

**FIGURE 5:**
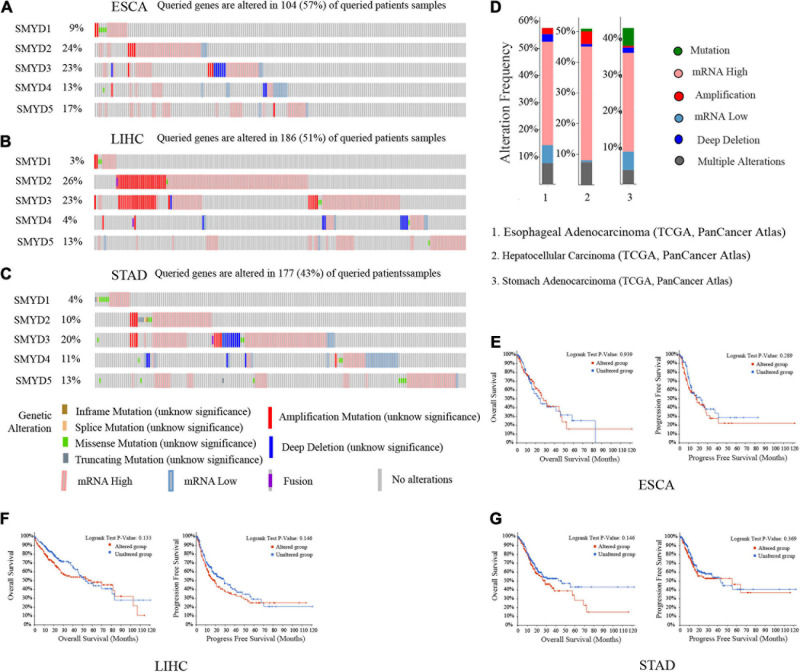
Mutation of SMYD family members in ESCA, LIHC, and STAD patients (cBioPortal). A visual summary of mutation frequency of SMYD family members **(A–C)**. Summary of mutation frequency of SMYD family members in ESCA, LIHC, and STAD patients **(D)**. Kaplan–Meier plotter was used to compare the relationship between gene mutation (red) and gene non-mutation (blue) of SMYD family members and OS and PFS **(E–G)** (*P* < 0.05 with statistical significance).

We analyzed SMYD family gene mutations and their relationship with OS and PFS in ESCA, LIHC, and STAD patients using the cBioPortal website and observed a high mutation frequency in SMYD genes. Among 181 ESCA patients, 104 had a mutation, with a mutation rate of 57%. The mutation rates of SMYD1/2/3/4/5 were 9, 24, 23, 13, and 17%, respectively. The mutation rate of SMYD2 was the highest and that of SMYD1 was the lowest. Among 366 LIHC patients, 186 had a mutation, with a mutation rate of 51%. The mutation rates of SMYD1/2/3/4/5 were 3, 26, 23, 4, and 13%, respectively. The mutation rate of SMYD3 was the highest and that of SMYD1 was the lowest. Among 412 STAD patients, 177 had a mutation, with a mutation rate of 43%. The mutation rates of SMYD1/2/3/4/5 were 4, 10, 20, 11, and 13%, respectively. The mutation rate of SMYD3 was the highest and that of SMYD1 was the lowest (Figures 5A–C). High SMYD mRNA expression was an important factor leading to high mutation frequency in ESCA, LIHC, and STAD (Figure 5D). However, Kaplan–Meier plotter and log-rank test analysis showed that SMYD family mutations had no significant correlation with OS and PFS in ESCA, LIHC, and STAD patients (OS, *P* = 0.939, *P* = 0.133, *P* = 0.146; PFS, *P* = 0.289, *P* = 0.146, *P* = 0.369) (Figures 5E–G). Next, we used the cBioPortal to search for the top 10 ESCA, LIHC, and STAD genes that are significantly related to SMYD family gene mutations (Table 2). After deduplication, a total of 124 genes were obtained.

**TABLE 2:** The top 10 ESCA, LIHC, and STAD genes significantly associated with SMYD family mutations (cBioPortal).

	ESCA	LIHC	STAD
SMYD1	HAND2-AS1, ADAMTS19, SLC2A4, SCRG1, TCEAL2, SORBS1, ACTG2, FNDC5, HSPB7, SYNPO2	FRMD7, CYP2A13, TGIF2LX, DDX53, TGIF2LY, MAGEB6, NUDT16P1, SIKE1, LOC391343, MNS1	ASB5, CHRM2, HAND2-AS1, DES, ACTG2, HAND2, PLIN4, CHRNA3, CNN1, LMO1
SMYD2	DTL, NVL, INTS7, ENAH, KIF14, SNAP47, TBCE, PPFIA4, FA2H, NUCKS1	RHBG, GLUL, GNPAT, LGR5, CDK6, ZNRF3, HPGD, C1QTNF3, MAP3K8, INSIG2	TBCE, NEK2, WDR12, UCHL5, NUF2, ILF2, CCT3, PACC1, EXO1, LIN9
SMYD3	ACBD6, VPS37D, PYCR2, DUSP12, TSEN15, TFB2M, ZNF496, NRSN2, TMEM9, SV2A	CNIH4, RBM34, SNRPE, NVL, ABHD2, ACBD6, C1ORF35, VPS72, SSR2, PYCR2	ACBD6, VPS72, MRPL9, COMMD7, PIGC, UBE2Q1, PYCR2, SSR2, MRPS14, TBCE
SMYD4	RPA1, CNTROB, METTL16, WDR81, TOP3A, DVL2, KIAA0753, NEURL4, PRPF8, FXR2	RPA1, NCBP3, KIAA0753, SMG6, PRPF8, ZZEF1, RABEP1, VPS53, ANKFY1, PAFAH1B1	METTL16, NEURL4, NCBP3, KIAA0753, PRPF8, RPA1, SMG6, RABEP1, WDR81, VPS53
SMYD5	SECISBP2L, CCT7, TTC27, DHODH, TEDC2, TIMM50, SSC4D, FUS, PPM1G, C1ORF35	TPD52L2, G6PD, TAT, SCP2, MPV17, ATIC, HSD17B6, ALDH2, ALDH6A1, GYS2	RTKN, ERAL1, DDX56, WDR74, ADRM1, PCGF1, LYST, PPM1G, DTYMK, AUP1

### Functional Enrichment Analysis and PPI Network of SMYD Family Genes in ESCA, LIHC, and STAD Patients

**FIGURE 6:**
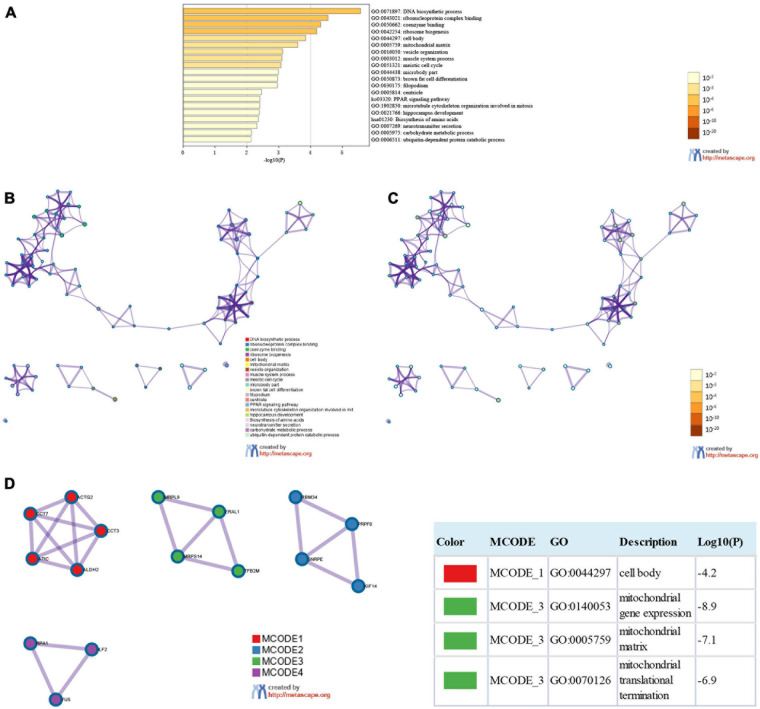
Enrichment analysis of SMYD family members and related mutant genes in ESCA, LIHC, and STAD (Metascape). Heat maps of GO and KEGG enrichment analysis of SMYD family members and 124 adjacent genes related to their mutations were stained with *P*-value **(A)**. Term-enriched network: colored by cluster ID, where nodes sharing the same cluster ID are usually close to each other **(B)**, colored by *P*-value, terms containing more genes tend to have more significant *P*-values **(C)**. For the MCODE components identified in the protein–protein interaction network, the three best score items divided by *P*-value are used as the functional description of the corresponding components, which are represented by the grid diagram **(D)**.

We used the 124 genes significantly related to SMYD family mutations for GO and KEGG enrichment analyses (Figures 6A–C). GO enrichment was divided into three functional groups: biological processes (11 items), molecular functions (two items), and cellular components (five items), and KEGG functional group (two items). We found that these genes were mainly involved in DNA biosynthesis, ribosome biogenesis, vesicle organization, muscle system process, meiotic cell cycle, brown fat cell differentiation, microtubule cytoskeleton organization involved in mitosis, hippocampus development, neurotransmitter secretion, carbohydrate metabolic process, ubiquitin-dependent protein catabolic process, PPAR signaling pathway, and amino acid biosynthesis. The molecular function of these genes is mediated via ribonucleoprotein complex and coenzyme binding. The cellular components involved in these genes were cell body, mitochondrial matrix, microbody part, filopodium, and centriole (Table 3). To better understand the relationship between SMYD family genes and ESCA, LIHC, and STAD, we conducted PPI network analysis. We performed enrichment analysis of pathways and processes for each MCODE component and found that the main component of the cells involved was the cell body, and the biological function was mainly related to mitochondrial gene expression, mitochondrial matrix, and mitochondrial translation (Figure 6D).

**TABLE 3:** Function enrichment analysis list of SMYD family members and adjacent genes Go and KEGG in ESCA, LIHC, and STAD (Metascape).

GO	Category	Description	Count	%	Log10(*P*)	Log10(*q*)
GO:0071897	GO biological processes	DNA biosynthetic process	8	6.50	–5.57	–1.21
GO:0043021	GO molecular functions	Ribonucleoprotein complex binding	6	4.88	–4.55	–0.89
GO:0050662	GO molecular functions	Coenzyme binding	8	6.50	–4.32	–0.74
GO:0042254	GO biological processes	Ribosome biogenesis	8	6.50	–4.19	–0.74
GO:0044297	GO cellular components	Cell body	10	8.13	–3.85	–0.72
GO:0005759	GO cellular components	Mitochondrial matrix	9	7.32	–3.60	–0.54
GO:0016050	GO biological processes	Vesicle organization	7	5.69	–3.13	–0.20
GO:0003012	GO biological processes	Muscle system process	8	6.50	–3.10	–0.19
GO:0051321	GO biological processes	Meiotic cell cycle	6	4.88	–3.07	–0.17
GO:0044438	GO cellular components	Microbody part	4	3.25	–2.99	–0.12
GO:0050873	GO biological processes	Brown fat cell differentiation	3	2.44	–2.97	–0.12
GO:0030175	GO cellular components	Filopodium	4	3.25	–2.96	–0.12
GO:0005814	GO cellular components	Centriole	4	3.25	–2.46	0.00
ko03320	KEGG pathway	PPAR signaling pathway	3	2.44	–2.41	0.00
GO:1902850	GO biological processes	Microtubule cytoskeleton organization involved in mitosis	4	3.25	–2.41	0.00
GO:0021766	GO biological processes	Hippocampus development	3	2.44	–2.39	0.00
hsa01230	KEGG pathway	Biosynthesis of amino acids	3	2.44	–2.36	0.00
GO:0007269	GO biological processes	Neurotransmitter secretion	4	3.25	–2.31	0.00
GO:0005975	GO biological processes	Carbohydrate metabolic process	8	6.50	–2.15	0.00
GO:0006511	GO biological processes	Ubiquitin-dependent protein catabolic process	8	6.50	–2.14	0.00

*It includes the first 16 clusters and their representative enrichment terms (one for each cluster). “Count” is the number of genes in the provided list that have membership in the given ontology term. “%” is the percentage of all genes provided found in a given ontology term (only input genes with at least one ontology term annotation are included in the calculation). “Log10(*P*)” is the *P*-value based on Log10. “Log10(*q*)” is a multi-test adjusted *P*-value based on Log10.*

### Correlation Between SMYD Family mRNA Expression in ESCA, LIHC, and STAD Patients With Immune Cell Infiltration and Immune Check Sites

**FIGURE 7:**
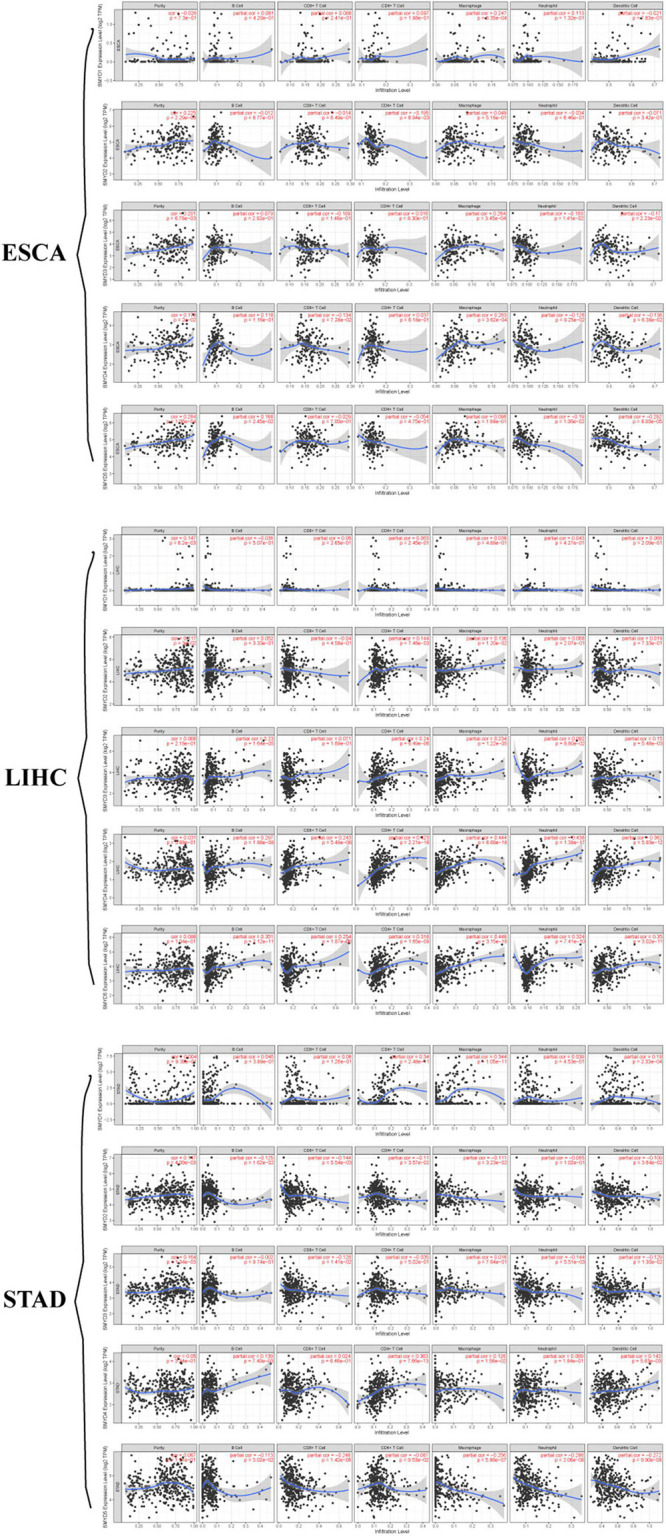
Correlation between SMYD members and immune cell infiltration, *X*-axis is SMYD members, *Y*-axis is immune infiltrates, *P*-value greater than 0 is positive correlation, *P*-value less than 0 is negative correlation. (Timer) (*P* < 0.05, with statistical significance).

**FIGURE 8:**
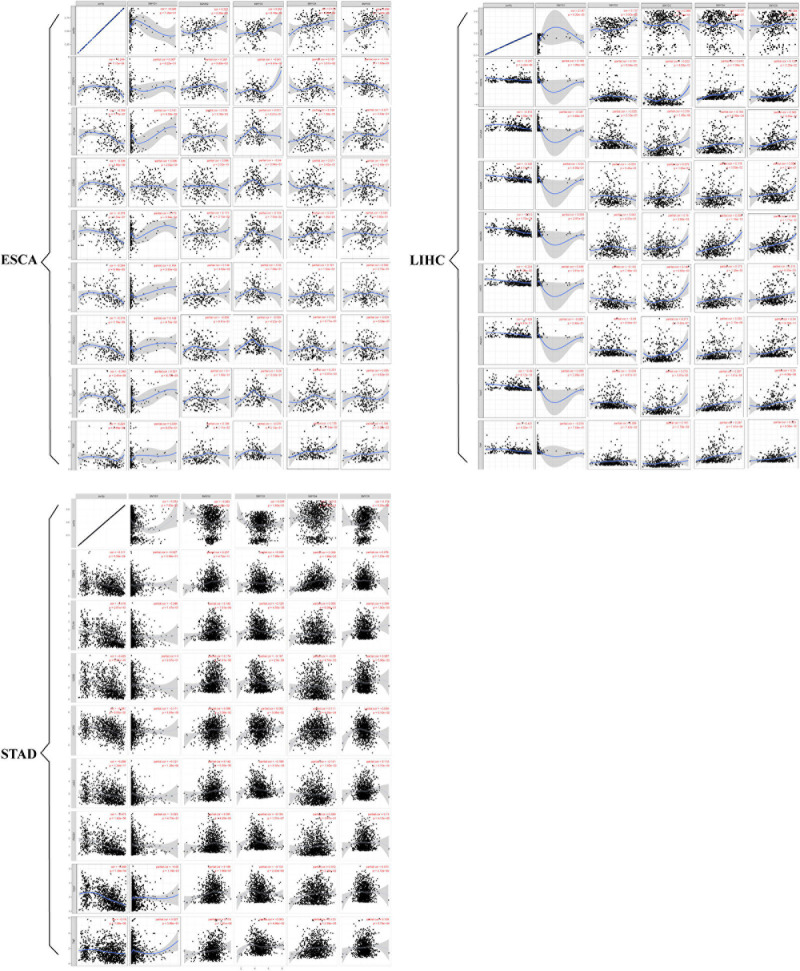
Correlation between SMYD members and immune cell biomarkers, *X*-axis is SMYD members, *Y*-axis is immune cell biomarkers, *P*-value greater than 0 is positive correlation, *P*-value less than 0 is negative correlation. (Timer) (*P* < 0.05 is statistically significant).

The prospect of immunotherapy is broad. PD-1 inhibitors are immunotherapeutic drugs used in the treatment of ESCA, LIHC, and GC. Therefore, we used the TIMER website to verify the correlation between the expression of SMYD family genes and immune cell infiltration and immune check sites in ESCA, LIHC, and STAD patients. We found that the mRNA expression of SMYD family members in ESCA, LIHC, and STAD patients was significantly correlated with six kinds of immune cells, including B cells, CD8+ T cells, CD4+ T cells, macrophages, neutrophils, and dendritic cells. Specifically, in ESCA, SMYD1 was positively correlated with macrophages, SMYD2 was negatively correlated with CD4+ T cells, and SMYD3 was positively correlated with macrophages and negatively correlated with neutrophils and dendritic cells. SMYD4 was positively correlated with macrophages, whereas SMYD5 was negatively correlated with neutrophils and dendritic cells (Figure 7 and Table 4). In LIHC, SMYD2 were positively correlated with CD4+ T and macrophages cells, SMYD3 was positively correlated with B cells, CD4+ T cells, macrophages, and dendritic cells. SMYD4/5 was positively correlated with B cells, CD8+ T cells, CD4+ T cells, macrophages, neutrophils, and dendritic cells (Figure 7 and Table 4). In STAD, SMYD1 was positively correlated with CD4+ T cells, macrophages, and dendritic cells, SMYD2 was negatively correlated with CD8+ T cells and dendritic cells, SMYD3 was negatively correlated with neutrophils, SMYD4 was positively correlated with B cells, CD4+ T Cells and dendritic cells, and SMYD5 was negatively correlated with CD8+ T cells, macrophages, neutrophils, and dendritic cells (Figure 7 and Table 4). After adjusting for tumor purity, we performed a correlation analysis between SMYD family members in ESCA, LIHC, and STAD patients, and eight common immune check sites and found significant correlations. Specifically, in ESCA, SMYD1 was positively correlated with HAVCR2 and TIGIT, and negatively correlated with LAG3 and PDCD1. SMAD2 was positively correlated with HAVCR2, LAG3, PDCD1, and TNF, whereas SMAD3 was negatively correlated with LAG3. SMAD4 was positively correlated with CD274, CTLA4, and HAVCR2, and SMAD5 was positively correlated with LAG3 and TNF (Figure 8 and Table 5). In LIHC, SMYD1 was positively correlated with CD274, SMAD2 was negatively correlated with LAG3, and SMAD3 was positively correlated with CTLA4, HAVCR2, LAG3, PDCD1, TIGIT, and TNF. SMAD4 was positively correlated with CD274, CTLA4, GZMB, HAVCR2, LAG3, PDCD1, TIGIT, and TNF, whereas SMAD5 was positively correlated with CD274, CTLA4, HAVCR2, LAG3, PDCD1, TIGIT, and TNF (Figure 8 and Table 5). In STAD, SMYD1 was negatively correlated with HAVCR2 and LAG3, SMAD2 was positively correlated with CD274, CTLA4, GZMB, HAVCR2, LAG3, PDCD1, TIGIT, and TNF, and SMAD3 was negatively correlated with CTLA4, GZMB, LAG3, PDCD1, TIGIT, and TNF. SMAD4 was positively correlated with CD274, HAVCR2, TIGIT, TNF, and negatively correlated with GZMB, LAG3, whereas SMAD5 was positively correlated with CD274, CTLA4, GZMB, LAG3, PDCD1, TIGIT, and TNF (Figure 8 and Table 5). These results confirm that the SMYD family plays an important role in the immune pathways of ESCA, LIHC, and STAD.

**TABLE 4:** Correlation between SMYD members and ESCA, LIHC, and STAD immune cell infiltration (TIMER).

**Description**	SMYD1		SMYD2		SMYD3		SMYD4		SMYD5	
	Correlation	*P*	Correlation	*P*	Correlation	*P*	Correlation	*P*	Correlation	*P*
B cell (ESCA)	0.061	4.20e-01	–0.012	8.77e-01	0.079	2.92e-01	0.118	1.16e-01	**0.168**	*
CD8+ T cell (ESCA)	0.088	2.41e-01	–0.014	8.49e-01	–0.109	1.46e-01	–0.134	7.28e-02	–0.029	7.00e-01
CD4+ T cell (ESCA)	0.097	1.95e-01	**−−0.195**	**	0.016	8.30e-01	0.037	6.18e-01	–0.054	4.75e-01
Macrophage (ESCA)	**0.247**	***	0.049	5.16e-01	**0.264**	***	**0.263**	***	0.096	1.98e-01
Neutrophil (ESCA)	0.113	1.32e-01	–0.034	6.46e-01	**−−0.183**	*	–0.126	9.25e-02	**−−0.19**	**
Dendritic cell (ESCA)	–0.021	7.83e-01	–0.071	3.42e-01	**−−0.17**	*	–0.138	6.38e-02	**−−0.292**	***
B cell (LIHC)	–0.036	5.07e-01	0.052	3.33e-01	**0.23**	***	**0.297**	***	**0.351**	***
CD8+ T cell (LIHC)	0.06	2.65e-01	–0.04	4.58e-01	0.071	1.89e-01	**0.243**	***	**0.254**	***
CD4+ T cell (LIHC)	0.063	2.45e-01	**0.144**	**	**0.24**	***	**0.423**	***	**0.318**	***
Macrophage (LIHC)	0.038	4.89e-01	**0.136**	**	**0.234**	***	**0.444**	***	**0.448**	***
Neutrophil (LIHC)	0.043	4.27e-01	0.068	2.07e-01	0.092	8.80e-02	**0.438**	***	**0.324**	***
Dendritic cell (LIHC)	0.068	2.09e-01	0.019	7.33e-01	**0.15**	**	**0.362**	***	**0.35**	***
B cell (STAD)	0.045	3.89e-01	–0.125	1.62e-02	–0.002	9.74e-01	**0.139**	**	–0.113	3.02e-02
CD8+ T cell (STAD)	0.08	1.26e-01	**−−0.144**	**	–0.128	1.41e-02	0.024	6.46e-01	**−−0.248**	***
CD4+ T cell (STAD)	**0.34**	***	–0.11	3.57e-02	–0.035	5.02e-01	**0.363**	***	–0.087	9.53e-02
Macrophage (STAD)	**0.344**	***	–0.111	3.23e-02	0.016	7.64e-01	0.126	1.56e-02	**−−0.256**	***
Neutrophil (STAD)	0.039	4.53e-01	–0.085	1.02e-01	**−−0.144**	**	0.069	1.84e-01	**−−0.286**	***
Dendritic cell (STAD)	**0.19**	***	**−−0.109**	*	–0.129	1.30e-02	**0.143**	**	**−−0.272**	***

Bold values indicated that the results were statistically significant (*P < 0.05, **P < 0.01, ***P < 0.001).

**TABLE 5:** Correlation between SMYD members and ESCA, LIH, and STAD immune cell biomarkers (TIMER).

Description	SMYD1		SMYD2		SMYD3		SMYD4		SMYD5	
	Correlation	*P*	Correlation	*P*	Correlation	*P*	Correlation	*P*	Correlation	*P*
CD274 (ESCA)	0.007	9.22e-01	**0.205**	**	–0.001	9.91e-01	**0.157**	*	0.104	1.63e-01
CTLA4 (ESCA)	0.151	4.30e-02	0.158	3.38e-02	0.031	6.81e-01	**0.199**	**	0.077	3.04e-01
GZMB (ESCA)	0.096	2.02e-01	0.096	2.02e-01	0.04	5.84e-01	0.071	3.42e-01	0.087	2.45e-01
HAVCR2 (ESCA)	**0.173**	*	**0.172**	*	0.133	7.53e-02	**0.231**	**	0.044	5.60e-01
LAG3 (ESCA)	**−−0.294**	***	**0.154**	*	**−−0.148**	*	–0.02	7.88e-01	**0.181**	*
PDCD1 (ESCA)	**−−0.319**	***	**0.148**	*	0.006	9.41e-01	0.059	4.32e-01	0.042	5.77e-01
TIGIT (ESCA)	**0.201**	**	0.1	1.80e-01	0.05	5.03e-01	0.251	5.03e-01	0.055	4.63e-01
TNF (ESCA)	0.039	6.07e-01	**0.189**	*	–0.076	3.13e-01	0.135	7.04e-02	**0.166**	*
CD274 (LIHC)	**0.166**	**	0.101	6.04e-02	–0.022	6.83e-01	**0.412**	***	**0.123**	*
CTLA4 (LIHC)	–0.047	3.88e-01	–0.035	5.16e-01	**0.318**	***	**0.184**	***	**0.362**	***
GZMB (LIHC)	0.04	4.59e-01	–0.033	5.43e-01	0.075	1.63e-01	**0.116**	*	0.066	2.21e-01
HAVCR2 (LIHC)	0.056	2.87e-01	0.042	4.37e-01	**0.16**	**	**0.338**	***	**0.388**	***
LAG3 (LIHC)	0.046	3.91e-01	**−−0.143**	**	**0.146**	**	**0.173**	**	**0.213**	***
PDCD1 (LIHC)	–0.063	2.40e-01	0.04	4.54e-01	**0.271**	***	**0.252**	***	**0.34**	***
TIGIT (LIHC)	0.056	2.96e-01	–0.039	4.67e-01	**0.219**	***	**0.297**	***	**0.29**	***
TNF (LIHC)	–0.016	7.64e-01	0.096	7.42e-02	**0.161**	**	**0.287**	***	**0.323**	***
CD274 (STAD)	–0.027	3.98e-01	**0.207**	***	–0.008	7.98e-01	**0.309**	***	**0.079**	*
CTLA4 (STAD)	–0.046	1.47e-01	**0.182**	***	**−−0.129**	***	0.008	8.08e-01	**0.099**	**
GZMB (STAD)	0	9.97e-01	**0.174**	***	**−−0.187**	***	**−−0.09**	**	**0.087**	**
HAVCR2 (STAD)	**−−0.171**	***	**0.098**	**	0.052	9.96e-02	**0.111**	***	–0.059	6.10e-02
LAG3 (STAD)	**−−0.121**	***	**0.142**	***	**−−0.168**	***	**−−0.101**	**	**0.112**	***
PDCD1 (STAD)	–0.023	4.73e-01	**0.091**	**	**−−0.165**	***	0.029	3.67e-01	**0.13**	***
TIGIT (STAD)	–0.05	1.16e-01	**0.165**	***	**−−0.133**	***	**0.072**	*	**0.073**	*
TNF (STAD)	0.027	3.96e-01	**0.178**	***	**−−0.063**	*	**0.133**	***	**0.109**	***

Correlation adjusted by purity. Bold values indicated that the results were statistically significant (*P < 0.05, **P < 0.01, ***P < 0.001).

## Discussion

It has been established that the SMYD gene family plays an important role in tumors. However, the mechanism underlying the function of different SMYD family members in malignant digestive system tumors remains largely unknown. We conducted a comprehensive analysis of mRNA expression differences, clinical stage and TP53 correlations, prognostic value, mutation, functional enrichment analysis, PPI analysis, immune cell infiltration, and correlation of immune check sites using mining and analysis of major online database websites, to explore the role of SMYD family members in malignant digestive system tumors. To the best of our knowledge, this is the first comprehensive analysis of the prognostic value of SMYD family members in malignant digestive system tumors and their relationship with immune infiltration.

Each SMYD family member has its own unique structural domain, cell functions and tissue distribution. A large number of studies have found that the SMYD family mainly regulates the transcription and translation of oncogenes or tumor suppressor genes, affecting tumor transcription regulation, chromosome remodeling, DNA damage repair, and signal transduction by: (1) promoting histone methylation, forming a transcription mplex with RNA polymerase II, specifically recognizing the promoter region of target genes, and promoting the transcriptional activation of downstream target genes, and (2) promoting non-histone protein methylation, directly binding with molecular chaperones, regulating key tumor signaling pathways and downstream target genes, and affecting the malignant characteristics of tumors (Carr et al., 2017).

SET and MYND domain-containing 1 protein, a regulator of heart and skeletal muscle, is the most unique SMYD family member. Subtypes A and B are expressed in striated muscle, while subtype C is expressed in CD8+ cells (Hwang and Gottlieb, 1997; Gottlieb et al., 2002). SMYD1 can be combined with muscle-specific transcription factor (skNAC) as a molecular chaperone to regulate histone H3K4 methylation, thereby playing a key role in ventricular cardiomyocyte expansion and regulation of skeletal muscle growth and regeneration (Park et al., 2010; Berkholz et al., 2015). The biological function of SMYD1 in tumors has not been investigated. We found that SMYD1 mRNA expression was significantly lower in GC than in normal tissues, whereas its expression in LIHC tissues was significantly higher than that in normal tissues. However, no significant expression difference was observed between ESCA and normal tissues. Clinical stage correlation analysis showed that SMYD1 mRNA expression in STAD stage-1 patients was significantly lower than that of stage-2/3. TP53 mutation correlation analysis showed that the mRNA expression of the TP53 non-mutated group was significantly increased in STAD. Survival analysis showed that SMYD1 was significantly correlated with RFS in ESCA patients, and with OS and RFS in STAD patients, suggesting that SMYD1 may be a potential tumor marker in these patients.

Increased SMYD2 expression has been significantly associated with the low survival rate of patients with esophageal squamous cell carcinoma, revealing the carcinogenic potential of SMYD2 (Komatsu et al., 2009). In BC, SMYD2, and p300/CAMP mediate estrogen receptor-α (Erα) methylation and acetylation, respectively, to form dynamic interactive regulation, affecting the transcriptional regulation of Erα (Zhang et al., 2013). In pancreatic ductal adenocarcinoma, SMYD2 promotes tumor formation by promoting the methylation of Lys355 of human mitogen-activated protein kinase activated protein kinase 3 (MAPKAPK3) (Reynoird et al., 2016). TP53, a tumor suppressor gene, is one of the few non-histone proteins regulated by lysine methylation (Levine, 1997; Vogelstein et al., 2000). Previous reports showed that SMYD2 overexpression promotes the methylation of Lys370 in p53 and inhibits p53-mediated transcriptional regulation, leading to cancer occurrence (Huang et al., 2006). We found high SMYD2 mRNA expression in LIHC in five studies in the Oncomine database, two of which showed high and one showed low SMYD2 mRNA expression in GC. TIMER and UALCAN database analyses showed significantly high SMYD2 mRNA expression in ESCA, LIHC, and STAD patients. Thus, SMYD2 mRNA expression appears to be significantly high in ESCA, LIHC, and STAD patients. Clinical stage correlation analysis showed that SMYD1 mRNA expression in stage-2 ESCA patients was significantly higher than that of stage-1/3, and that in LIHC stage-4 patients was significantly higher than that of stage-3. Correlation analysis of TP53 mutation showed that SMYD2 mRNA expression in the TP53 mutation group was significantly increased in ESCA and STAD patients, suggesting that the overexpression of SMYD2 mRNA may be related to TP53 mutation. Survival analysis showed that the OS and PFS of ESCA patients with high SMYD2 expression were significantly prolonged, unlike what previous studies have shown. This needs to be verified in a larger sample (Komatsu et al., 2009). The OS of patients with SMYD2 overexpression in LIHC was significantly shortened.

SET and MYND domain-containing 3 may play an important role in the occurrence and development of tumors. Especially in BC, high SMYD3 expression promotes tumor cell proliferation. Downregulation of SMYD3 expression induces G1 phase cell cycle arrest and subsequent apoptosis (Ren et al., 2011). Furthermore, high SMYD3 expression promotes BC occurrence by directly regulating the expression of the proto oncogene WNT10B (Hamamoto et al., 2006). Previous studies showed that SMYD3 is an ER-mediated transcriptional coactivator, which can enhance the ER receptor’s ligand response, closely related to BC (Kim et al., 2009). In colon cancer and hepatocarcinoma cell lines, SMYD3 forms transcription complexes with HSP90 and RNA polymerase II to promote H3K4 methylation and regulate the transcription of the target gene NK2homeobox8 (Nkx28), promoting tumor cell proliferation. Similar results have been obtained in cervical cancer cell line models. Downregulation of SMYD3 expression significantly reduces the ability of tumor cells to expand and migrate *in vitro* (Wang et al., 2008). In GC, studies have shown that high SMYD3 expression promotes GC cell proliferation, migration, and invasion through ATM signaling, and that SMYD3 may become a therapeutic target for GC patients (Wang et al., 2017). We found a significantly high SMYD3 mRNA expression in ESCA, LIHC, and GC patients. Clinical correlation analysis showed that SMYD3 mRNA expression in ESCA stage-1 patients was significantly lower than that in stage-2/3. TP53 mutation correlation analysis showed that SMYD3 mRNA expression in the TP53 mutation group was significantly increased in ESCA, LIHC, and STAD patients, suggesting that SMYD3 mRNA overexpression may also be related to TP53 mutation. Survival analysis results showed that the RFS of patients with high SMYD3 expression in ESCA was significantly shortened, and that the OS and RFS of patients with SMYD3 overexpression in LIHC and STAD were significantly shortened. Consistent with our findings, previous studies showed that SMYD3 overexpression was an independent prognostic risk factor for poor prognosis in LIHC (Fei et al., 2017), that the expression of SMYD3 was significantly positively correlated with the expression of transcription 3 (STAT3), and that the prognosis of GC patients with high SMYD3 expression was poor (Liu Y. et al., 2015).

Cancer stem cells (CSC) are responsible for tumor development, metastasis and recurrence. SMYD4 is closely related to CSCs. It has been reported that SMYD4 binds to miR-135a and activates the expression of Nanog by regulating the methylation of its promoter, contributing to the conversion between CSCs to non-CSCs. SMYD4 has not been extensively studied in cancer. Only in BC, SMYD4 exerts anti-tumor effects through local inhibition of PDGFR-A (Hu et al., 2009). We found significant SMYD3 mRNA overexpression in ESCA, LIHC, and STAD patients. Clinical correlation analysis showed that SMYD4 mRNA expression in LIHC stage-3 patients was significantly higher than that in stage-1/2 patients, and that the mRNA expression of stage-3 STAD patients was significantly higher than that of stage-1 patients. Correlation analysis of TP53 mutation showed that SMYD3 mRNA expression in the TP53 mutation group was significantly increased in ESCA, LIHC, and STAD patients. Survival analysis showed that the OS and RFS of ESCA patients with SMYD4 overexpression were significantly shortened, and that the OS of LIHC and STAD patients with SMYD4 overexpression was significantly shortened. However, the role of SMYD4 in malignant digestive system tumors requires further investigation.

SET and MYND domain-containing 5 has been identified as a key regulator of BC cell cancer metastasis. SMYD5 inhibits Toll-like receptor 4 (TLR4) expression in macrophages through H4K20me3, thereby regulating immune system balance (Stender et al., 2012). We found significantly high SMYD5 mRNA expression in ESCA, LIHC, and STAD patients. Clinical correlation analysis showed that SMYD5 mRNA expression in LIHC stage-1 patients was significantly higher than that in stage-2/3. Correlation analysis of TP53 mutation showed that SMYD3 mRNA expression in the TP53 mutation group was significantly increased in ESCA, LIHC, and STAD patients. Survival analysis showed that the OS of ESCA patients with SMYD5 overexpression was significantly prolonged. The OS and RFS of LIHC patients with SMYD5 overexpression were significantly prolonged, indicating that SMYD5 may be a potential tumor marker and therapeutic target for immune and targeted therapy in ESCA and LIHC patients.

High SMYD family mutation frequency was found in ESCA, LIHC, and STAD patients, with total mutation rates of approximately 57, 51, and 43%, respectively. Previous studies have shown that SMYD3 expression is increased in KRAS-mutated cancer, which may be due to the regulative effect of KRAS on SMYD3 gene transcription or protein stability. SMYD3 silencing may reduce the progression of advanced cancer rendering SMYD3 a potential therapeutic target for cancer patients with KRAS mutations (Mazur et al., 2014). Using GO and KEGG enrichment analyses and PPI network analysis of 124 genes significantly related to SMYD family mutations, we found that their biological functions were mainly achieved through mitochondrial gene expression, mitochondrial matrix, mitochondrial translation, and other pathways. The complex regulatory mechanisms between these molecular pathways and tumor cell proliferation, invasion, migration, and epithelial to mesenchymal transition, require further investigation.

The SMYD family is also closely related to immune infiltration. Xu et al.’s (2015) research showed that SMYD2 is a novel negative regulator of macrophage activation and M1 polarization. Its high expression inhibits the production of pro-inflammatory cytokines including IL-6 and TNF, and inhibits the expression of important cell surface molecules. Furthermore, macrophages with high SMYD2 expression inhibit Th-17 cell differentiation and promote regulatory T cell differentiation (Xu et al., 2015). Nagata et al.’s (2015) research showed that SMYD3 regulated the expression of Foxp3 through a mechanism that relied on TGFβ1/SMYD3, thereby activating the formation of Treg cells. Stender et al.’s (2012) research showed that SMYD5 methylates H4 K20 and regulates the expression of TLR4-target genes, such as CXCl10, IL1a, and CCL4. We found that SMYD family members are closely related to six types of immune cells and eight immune check sites, which may provide insight into improving ESCA, LIHC, and GC immunotherapy. Thus, we believe that the SMYD family will play a central role in immunotherapy research, leading to important future discoveries.

Our study has some limitations, such as the use of database-retrieved data, lack of real world verification using cell, animal, and tissue studies, or investigations of the relevant underlying molecular mechanism and clinical application in ESCA, LIHC, and GC treatment. In conclusion, our study identified a high frequency of SMYD family mutations and differential SMYD family gene expression in malignant digestive system tumors, indicating that the SMYD family may provide potential prognosis prediction markers, and immune or targeted therapy targets.

## Data Availability Statement

Publicly available datasets were analyzed in this study. This data can be found here: www.oncomine.org; http://ualcan.path.uab.edu/analysis.html; www.kmplot.com; www.cbioportal.org; http://metascape.org; https://cistrome.shinyapps.io/timer/.

## Ethics Statement

Ethical review and approval was not required for the study on human participants in accordance with the local legislation and institutional requirements. Written informed consent for participation was not required for this study in accordance with the national legislation and the institutional requirements.

## Author Contributions

DL and MN: conceptualization. QJ and PK: methodology. SS and CW: formal analysis. SY and ZQ: investigation. DL and SZ: writing – original draft preparation. XW: writing – review and editing. ES: supervision; LW: project administration. LL: funding acquisition. All authors have read and agreed to the published version of the manuscript.

## Conflict of Interest

The authors declare that the research was conducted in the absence of any commercial or financial relationships that could be construed as a potential conflict of interest.

## Footnotes

1 www.oncomine.org

2 http://ualcan.path.uab.edu/analysis.html

3 www.kmplot.com

4 www.cbioportal.org

5 http://metascape.org

6 https://cistrome.shinyapps.io/timer/
