# Supplementary figures and images for: Comprehensive Analysis of the Value of SMYD Family Members in the Prognosis and Immune Infiltration of Malignant Digestive System Tumors
Source: Front Genet. 2021 Jul 16;12:699910. doi: 10.3389/fgene.2021.699910 (PMC8322783; doi:10.3389/fgene.2021.699910)

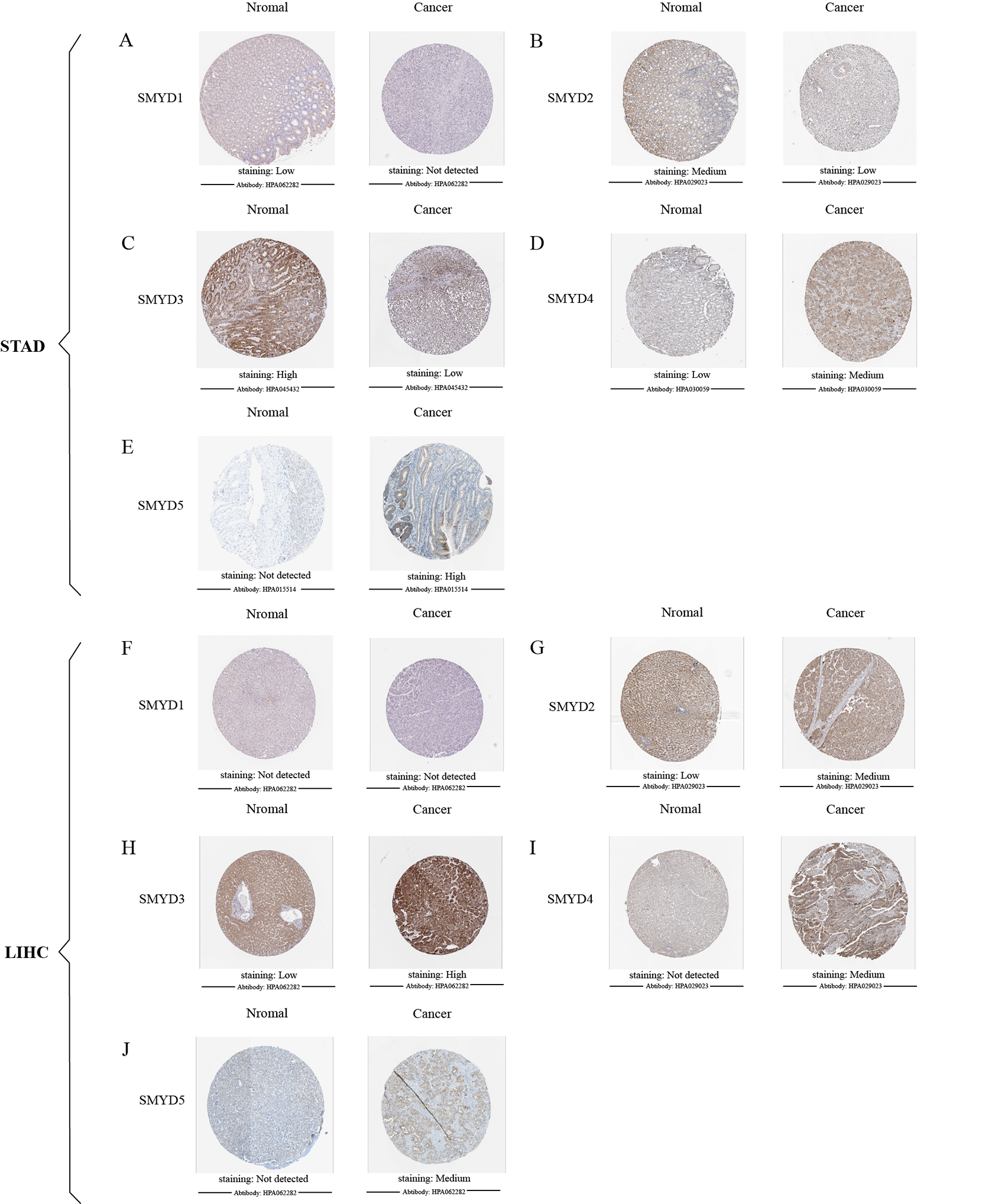

Supplement: Supplementary file 1 [file Image_1.TIF]
